# What Systems Teach: The Psychological Meaning of Delay in Youth Mental Health Access

**DOI:** 10.1192/j.eurpsy.2026.10572

**Published:** 2026-07-31

**Authors:** A. Isak

**Affiliations:** University of Vienna, Vienna, Austria

## Abstract

**Introduction:**

Delays in youth mental health care are well documented, but most research treats them as logistical barriers rather than lived experiences. Little is known about the implicit messages that delays communicate to young people about worthiness or priority, and how these meanings influence trust in services and future engagement.

**Objectives:**

To examine how young people interpret delays or unclear processes when seeking mental health support, and how these interpretations affect motivation, trust, and engagement.

**Methods:**

An anonymous online survey was conducted with participants aged 18+, reflecting on help-seeking attempts between the ages of 14–25. The mixed-methods design included quantitative items on trust, fairness, and help-seeking persistence, together with open-ended questions on the meaning of the process. Descriptive statistics summarise quantitative responses, and thematic analysis is applied to qualitative data.

**Results:**

Preliminary findings suggest that delays and unclear processes reduce motivation to persist with help-seeking and can contribute to disengagement. Qualitative analysis highlights recurring narratives of feeling “not sick enough”, questioning the adequacy of services, and withdrawing to cope alone.

**Conclusions:**

Delays and unclear processes not only slow access — they also appear to convey implicit messages that shape young people’s sense of deservingness and their trust in care systems. Understanding these meanings is critical for youth-centred service redesign and mental health policy.

**Disclosure of Interest:**

None Declared

